# When imaging technology meets single-cell omics: new paradigm in developmental biology

**DOI:** 10.1186/s13619-025-00276-4

**Published:** 2025-12-17

**Authors:** Chunling Wang, Xuejing Zhang, Yifan Zhang, Feng Liu

**Affiliations:** 1 Shandong Provincial Key Laboratory of Animal Cell and Developmental Biology, School of Life Sciences, Qilu Hospital (Qingdao), Cheeloo College of Medicine, Shandong University, Qingdao, 266237 China; 2 State Key Laboratory of Organ Regeneration and Reconstruction, Beijing Institute for Stem Cell and Regenerative Medicine, Institute of Zoology, University of Chinese Academy of Sciences, Chinese Academy of Sciences, Beijing, 100101 China

**Keywords:** Developmental biology, Imaging technologies, Single-cell omics, Spatial omics, Integration

## Abstract

Advanced imaging and single-cell omics technologies are fundamentally transforming developmental biology research, shifting it from static observation to dynamic, spatially resolved systems biology. Super-resolution microscopy breaks the diffraction barrier to visualize nanoscale subcellular dynamics, while light-sheet fluorescence microscopy enables long-term, multi-scale volumetric imaging of living specimens. In parallel, single-cell omics (e.g., transcriptomics and proteomics) decipher molecular heterogeneity and lineage trajectories, and spatially resolved transcriptomics maps gene expression within native tissue contexts at subcellular resolution. However, each approach has inherent limitations: imaging lacks deep molecular profiling, while dissociation-based omics loses spatial context. This review highlights how the integration of these technologies bridges cellular behaviors with molecular mechanisms, providing unprecedented multi-scale perspectives on key developmental processes—including embryogenesis, organogenesis, neural patterning, and disease progression. By synergistically capturing the "when," "where," and "how" of developmental processes, this convergence resolves longstanding questions and establishes a new mechanistic and predictive paradigm in developmental biology.

**Graphical Abstract:**

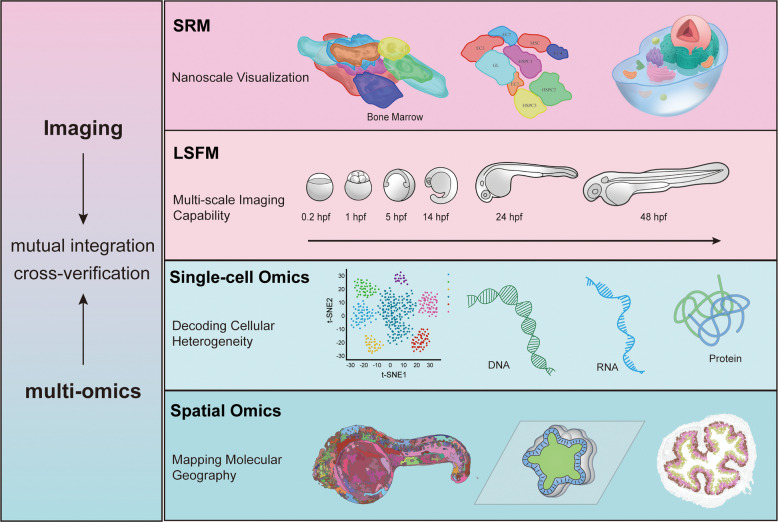

## Background

Developmental biology is undergoing a fundamental transformation. Classical approaches, such as descriptive embryology and bulk molecular analyses, have provided foundational insights into cell fate decisions, lineage commitment, tissue patterning, and functional maturation. However, these traditional methods fall short of capturing the spatial and temporal complexity of molecular dynamics in their native tissue contexts. A critical barrier remains: the inability to resolve cellular heterogeneity and molecular interactions across scales while preserving spatiotemporal information.

Developmental biology has advanced through successive technological waves that systematically overcame prior constraints. The molecular genetics revolution of the 1980s–90s, enabled by mutagenesis screens, positional cloning, and early confocal imaging, first linked genes to developmental patterning. However, these approaches were limited to low-throughput, population-level snapshots lacking cellular resolution. The genomic era of the 2000s enabled systematic network reconstruction, but remained blind to single-cell heterogeneity and spatial dynamics (Liberali and Schier 2024). Since the 2010s, the convergence of single-cell omics and advanced imaging (super-resolution, light-sheet) has captured dynamic molecular states and cellular behaviors across entire embryos at single-cell resolution while preserving spatial context. This historical trajectory, from linear gene discovery to comprehensive network mapping, establishes the foundation for integrating these technologies to decode how molecular programs orchestrate organismal form.

Recently, the convergence of advanced imaging technologies—such as super-resolution microscopy (SRM) and light-sheet fluorescence microscopy (LSFM) with single-cell omics approaches, including single-cell and spatial transcriptomics, has initiated a new paradigm: spatiotemporally resolved systems biology. This synergy enables researchers to simultaneously monitor dynamic cellular behaviors and molecular states, overcoming the limitations of either modality alone. It allows for the precise mapping of molecular heterogeneity and lineage trajectories while visualizing real-time cellular dynamics during development.

This review explores how the integration of imaging and single-cell omics is resolving longstanding challenges in developmental biology. We highlight representative studies across key processes—including embryogenesis, organogenesis, neurodevelopment, hematopoiesis, and disease modeling—that exemplify the power of this combined approach. Ultimately, this fusion is transforming developmental biology from a descriptive discipline into a mechanistic and predictive science.

## Visualizing cross-scale dynamics: live imaging from cells to organisms

The innovation of imaging technology is the core driving force that has propelled developmental biology into an era of dynamic and systematic research. These efforts are primarily focused on two complementary technical directions: to break the spatial resolution limit with SRM for resolving subcellular structures at the nanoscale, and to employ LSFM for long-term, low-photodamage imaging of multiscale developmental processes in living specimens. The combined application of these techniques enables a new imaging paradigm that achieves both high spatial resolution and dynamic visualization in living systems. These advances now allow for a high-resolution, dynamic panorama of developmental processes within living organisms, spanning large scales and extended durations.

The diffraction limit of classical optical microscopy (~ 200 nm) (Abbe 1873) obscures numerous critical nanoscale developmental processes. SRM breaks the diffraction limit, achieving a resolution of 10–100 nm through innovations in both physical principles and computational methods (Table 1). The illumination-field modulation, such as stimulated emission depletion (STED) (Vicidomini et al. 2018) and structured illumination microscopy (SIM) (Gustafsson 2000), which use patterned light to enhance resolution to 20 nm. In contrast, single-molecule localization methods like stochastic optical reconstruction microscopy (STORM) (Rust et al. 2006) and photoactivated localization microscopy (PALM) (Betzig et al. 2006) achieve exceptional resolution below 20 nm by sequentially activating and localizing individual fluorescent molecules. Recently, deep learning approaches have further accelerated SRM by enabling super-resolved image reconstruction from conventional datasets (Wang et al. 2025).

**Table 1:** Comparison of Major Super-Resolution Microscopy Techniques

Technique	Type	Resolution	Key Principle	Application
STED	Illumination-field	~ 20 nm	Depletion of outer region of PSF	Live-cell organelle imaging
SIM	Illumination-field	~ 100 nm	Patterned illumination & reconstruction	Cytoskeleton dynamics
STORM/PALM	Single-molecule	< 20 nm	Stochastic activation & localization	Chromatin organization
Deep Learning-Based SRM	Computational	~ 50–100 nm	AI-enhanced image reconstruction	Mitochondrial autophagy

The value of SRM extends far beyond resolving static structures, enabling real-time observation of previously inaccessible dynamic processes. For example, the combination of STORM and SIM has revealed dynamic remodeling of vimentin intermediate filaments during B-cell activation in mouse spleen (Tsui et al. 2018), while deep learning-enhanced SIM has captured mitophagy events during stem cell differentiation at 60 nm resolution, providing visual evidence for metabolic reprogramming underlying cell fate transitions (Qiao et al. 2023).

**Fig. 1:**
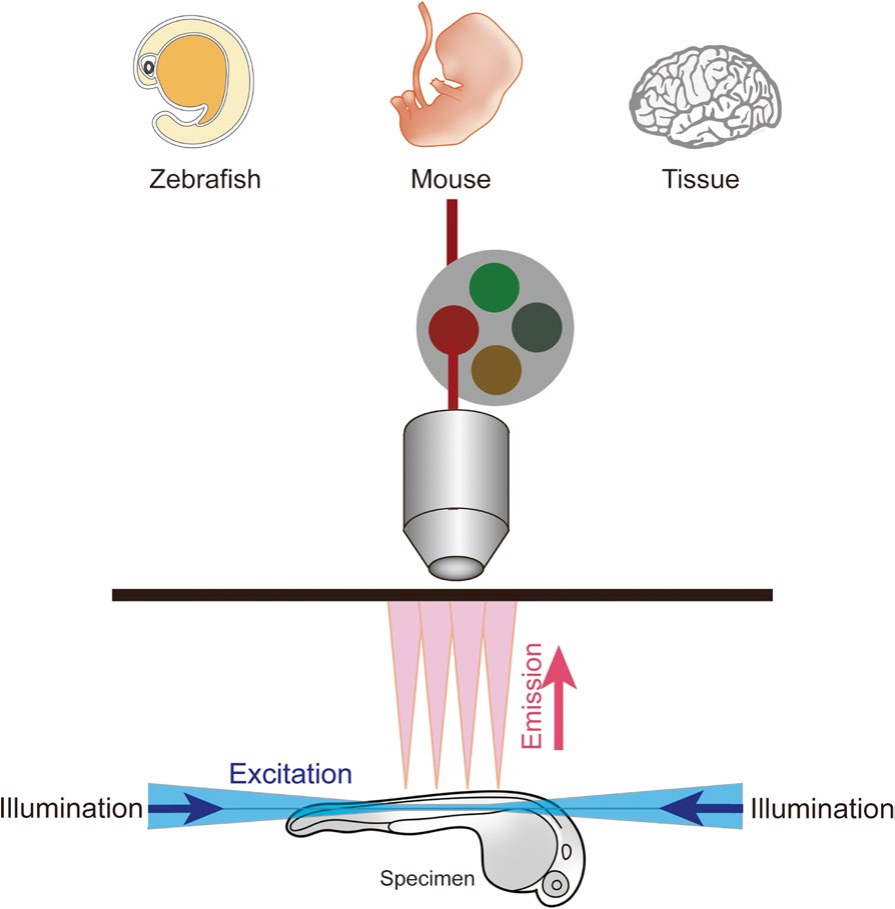
Principle and Application of Light-Sheet Fluorescence Microscopy (LSFM) in Developmental Biology. This schematic illustrates the working principle of LSFM, which uses a thin sheet of laser light to illuminate only the focal plane of the sample, minimizing phototoxicity and enabling rapid, high-resolution volumetric imaging of live specimens. LSFM supports multi-scale imaging from whole embryos to subcellular structures, facilitating long-term observation of dynamic developmental processes such as embryogenesis, organ formation, and tissue remodeling. Applications include lineage tracing in zebrafish, 3D reconstruction of human embryonic structures, and analysis of immune cell dynamics in intact organs

Except nanoscale imaging, embryonic development involves coordinated changes across spatiotemporal scales, from subcellular trafficking to whole-organism morphogenesis. Conventional confocal microscopy is limited in long-term in vivo observation due to its high phototoxicity and slow three-dimensional acquisition rate. LSFM addresses these limitations by illuminating only the imaging plane, enabling rapid, volumetric imaging with minimal photodamage (Chen et al. 2022a; Raju et al. 2025). Its technical scalability supports multi-scale studies ranging from whole embryos to specific organs (Fig. 1): At the organism level, LSFM allows non-invasive imaging of zebrafish throughout gastrulation to the emergence of organ primordia, facilitating cell lineage tracing and morphogenetic analysis (Daetwyler et al. 2025). At the organ level, it enables high-resolution 3D imaging of immune cell distribution and vascular networks within intact murine brains and hearts (Zhang et al. 2024). Integration with serial block-face scanning electron microscopy (CLEM-SBFSEM) has provided ultrastructural insights into hematopoietic stem/progenitor cells (HSPCs) in zebrafish kidney marrow (Agarwala et al. 2022). Combined with tissue clearing techniques (e.g., 3DISCO), LSFM further contributes to reconstructing 3D atlases of complex structures such as the peripheral nervous system in early human embryos (Belle et al. 2017).

The integration of SRM and LSFM represents an emerging trend in imaging technology development. For instance, adaptive SRM-light-sheet platforms (e.g., Meta-rLLS-VSIM) have further enabled near-isotropic 150 nm resolution at 1 Hz in live zebrafish embryos, facilitating minimally invasive tracking of organelle interactions during early development (Qiao et al. 2025). Such integrated technology bridges the observational gap between nanoscale static structures and dynamic functions in living systems, providing a powerful tool for uncovering the relationship between cellular behavior and molecular mechanisms within authentic developmental contexts.

## Decoding cellular heterogeneity: single-cell omics in developmental trajectories

**Fig. 2:**
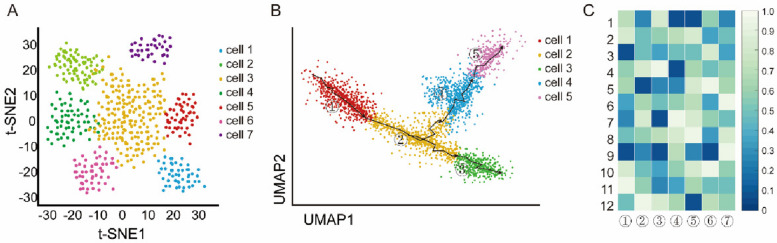
Uncovering Cellular Heterogeneity and Lineage Trajectories with scRNA-seq. **A** Uniform Manifold Approximation and Projection (t-SNE) plot of single-cell transcriptomes. **B** Inferred lineage trajectories(UMAP). **C** Dynamic gene expression along pseudotime.

Cellular heterogeneity is the engine of development, driving fate decisions from pluripotency to specialized function. Single-cell omics technologies, particularly single-cell RNA sequencing (scRNA-seq), allow for comprehensive profiling of gene expression in thousands of individual cells, revealing novel cell types and differentiation pathways (Tang et al. 2009) (Fig. 2).

In mouse embryogenesis, scRNA-seq enabled precise cell type classification and delineation of lineage specification, mapping a detailed atlas with over 500 cell subtypes and 56 developmental trajectories spanning E9.5–E13.5 (Cao et al. 2019), and further defined cell states across 19 successive stages from E3.5 to E13.5 (Qiu et al. 2022). Applied to the study of cell fate diversification in embryogenesis, scRNA-seq identified hematopoietic lineage bias in mesodermal cells during early human development (Wen et al. 2024). It also verified that macrophages originated from CD44^+^ endothelial cells in mouse placenta (Liang et al. 2021). In addition to normal development, scRNA-seq further delineates cell type-specific transcriptional profiles in early-stage lung fibrosis (Lang et al. 2023) and familial tauopathy pathogenesis (Sirkis et al. 2023), thereby offering unprecedented molecular insights into disease progression through a single-cell lens.

Beyond transcriptomics, single-cell proteomics by mass spectrometry (SCoPE-MS) has quantified protein expression during stem cell differentiation at distinct time points following differentiation induction (Budnik et al. 2018), and single-cell DNA sequencing (scDNA-seq) has revealed clonal evolution patterns in leukemia (Morita et al. 2020). Single-cell metabolomics complements these approaches by profiling metabolic states and identifying disease-associated alterations (Feng et al. 2022). Furthermore, Single-cell multi-omics approaches, integrating transcriptomics, methylation, and chromatin accessibility data, have illuminated early germ layer specification. For instance, distinct epigenetic landscapes emerge in ectodermal and mesodermal lineages during gastrulation (Argelaguet et al. 2019).

Together, these tools dissect the molecular logic of development, from lineage bifurcation to pathological divergence, offering high-resolution snapshots of cellular identity and trajectory.

## Mapping molecular geography: spatially resolved omics technologies

Spatial context is essential for understanding development, as morphogenesis depends on positional cues and cell–cell interactions. While single-cell omics has been pivotal in revealing cellular heterogeneity, its dissociation-based methods erase spatial information, blurring tissue patterning cues and cell–cell communication networks essential for morphogenesis. Spatially resolved omics technologies overcome this by anchoring molecular data to native tissue coordinates, enabling direct correlation of gene expression with cellular microenvironment.

Spatially resolved transcriptomics technologies, such as slide-seq and spatial enhanced resolution omics-sequencing (Stereo-seq), utilize spatially resolved whole-transcriptome analysis based on next-generation sequencing (NGS). Slide-seq employs arrays of polystyrene beads (~ 10 μm in diameter), achieving single-cell resolution at the ~ 10-μm level (Rodriques et al. 2019; Stickels et al. 2021). In contrast, Stereo-seq uses spatial barcoding to attain subcellular resolution (≤ 500 nm), enabling gene expression mapping within tissue microenvironments. For example, several studies including Wang et al. (computational modeling, E6.5–E7.5) (Wang et al. 2023), Xie et al. (Stereo-seq, E7–E8) (Xie et al. 2025), and Kumar et al. (Slide-seq, E8.5–E9.5) (Sampath Kumar et al. 2023) have employed distinct approaches to map mouse embryonic and early organ development at single-cell resolution. In human embryo development, spatial transcriptomics-based 3D reconstruction of a gastrulating human embryo displayed the gene expression patterns, positional register and cell subtypes in different spatial position (Xiao et al. 2024). Stereo-seq has also mapped the spatiotemporal transcriptome of the mouse placenta from E7.5 to E14.5 (Wu et al. 2024). Furthermore, Stereo-seq studies in zebrafish have revealed the spatial developmental trajectory of early embryos and constructed a high-resolution spatiotemporal dynamic map of cardiac regeneration (Li et al. 2025b).

Moreover, the multiplexed error-robust fluorescence in situ hybridization (MERFISH) identifies numerous RNA species by employing multiple rounds of sequential hybridization and imaging, where image registration using fluorescent beads as fiducial markers achieves a final alignment error of ~ 20 nm (Chen et al. 2015). Owing to its ultra-high resolution, MERFISH has been employed to resolve the spatial architecture of complex tissue structures such as synapses and neurons. For instance, MERFISH has depicted molecular, cellular, and cytoarchitectural development across seven gestational stages and eight cortical regions in the human fetus, revealing early emergence of the six-layered neocortical structure and cortical areal specification (Qian et al. 2025), and intricate organization within the dorsal pons (Nardone et al. 2024). In disease progression studies, MERFISH has identified expression profiles, spatial positioning, and intercellular interactions of fibroblasts during murine colitis development (Cadinu et al. 2024). Additionally, it has resolved the zonal distribution of hepatocytes in fibrotic liver while spatially characterizing macrophage and mesenchymal subpopulations (Watson et al., 2025).

Multimodal platforms—such as those integrating scRNA-seq with chromatin accessibility or spatial technologies—further enrich spatial datasets with regulatory information. For example, the integration of target chromatin indexing and tagmentation (TACIT) with scRNA-seq enables charting of a single-cell-resolution epigenetic landscape from fertilization to blastocyst formation, revealing developmental heterogeneity and predicting cell fates (Wang et al. 2023). The integration of single-cell multi-omics, Visium, and CosMx generated spatial RNA expression profiles and single-nucleus accessible chromatin maps of human kidney cells in both healthy and pathological states (Abedini et al. 2024).

These spatial technologies provide a framework for interpreting single-cell data in anatomical context, transforming our understanding of how location influences identity and function during development (Table 2).

**Table 2:** Comparison of Spatially Resolved Transcriptomics Technologies

Technology	Resolution	Throughput	Key Feature	Ref
Slide-seq	~ 10 μm	High	Bead-based barcoding	Rodriques et al. 2019
Stereo-seq	≤ 500 nm	Very high	Subcellular barcoding	Wu et al. 2024
MERFISH	~ 20 nm	Moderate	Sequential FISH + barcoding	Qian et al. 2025
Xenium	~ 200 nm	High	In situ sequencing + imaging	Dennis et al. 2024

## Bridging scales: integrated technologies answering fundamental questions in developmental biology

Imaging technologies and single-cell omics platforms have traditionally offered complementary yet disconnected views of biological systems: the former capturing spatiotemporal dynamics with limited molecular breadth, and the latter providing deep molecular profiling devoid of native context. The critical advancement lies in moving beyond their parallel application to establishing a synergistic cycle of mutual guidance and validation. This integrated paradigm creates a virtuous feedback loop, where imaging directs omics to biologically significant questions and cell populations, and omics, in turn, provides the molecular targets that empower precise spatial and functional validation through imaging.

This integrative cycle often begins with imaging guiding omics. Spatial and dynamic observations pinpoint cells of interest, solving the problem of "where and when to look" within complex tissues. For instance, the Zebrahub resource utilizes LSFM-based lineage reconstruction to generate a spatiotemporal map that directly informs and guides the sampling strategy and interpretation of subsequent single-cell sequencing data, creating a comprehensive atlas of zebrafish development (Kim et al. 2025). Similarly, the strategic combination of in vivo lineage tracing with scRNA-seq and scATAC-seq was driven by the need to resolve hematopoietic endothelial cell (HEC) heterogeneity; the imaging-derived lineage history provided the essential framework for interpreting the cellular transitions revealed by omics (Xia et al. 2023). In mouse models, the prior establishment of genetic tracing codes creates a system where imaging-readable barcodes directly isolate specific lineages for high-throughput scRNA-seq, thereby guiding the omics analysis to elucidate lineage differentiation (Li et al. 2025a).

Conversely, the reciprocal flow of information—from omics to imaging—is fundamental for hypothesis testing and discovery. Omics analyses generate rich, unbiased molecular hypotheses that require spatial contextualization. In disease research, this "omics-first, imaging-second" pipeline is a cornerstone. A prime example is the study of congenital heart disease (CHD), where single-nucleus and single-cell RNA-seq first identified transcriptional signatures of abnormal cell states and a dysregulated immune microenvironment. These computational findings were then spatially validated and mapped using imaging mass cytometry (IMC), which confirmed the protein-level expression and precise tissue localization of the key signaling pathways predicted by the omics data (Hill et al. 2022). This pattern of using sequencing to discover a distinct immune landscape and then employing immunofluorescence for in situ confirmation was similarly applied in studies of dermatomyositis (Ye et al. 2022).

The integration of imaging and omics technologies has not only reshaped our understanding of embryogenesis, organogenesis, and disease, but has also fueled the development of high-resolution imaging-based omics research platforms. Xenium and CODEX integrates sequential fluorescence in situ hybridization with enzymatic decoding (ReadCoor/Cartana-derived) and high-resolution fluorescence microscopy. It achieves precise subcellular (200 nm) spatial localization of individual mRNA transcripts with high-throughput gene detection capability. The platform provides high-resolution histological imaging (hematoxylin&eosin staining or fluorescence markers), enabling integrated protein detection on the same tissue section. Crucially, it overlays spatial transcriptomic data with morphological images, enhancing cell typing and tissue architecture analysis. Moreover, Xenium is capable of characterizing subtle differential expression patterns of nuclear versus cytoplasmic RNA among subcellular populations within diverse human tissues—including human skeletal (To et al. 2024), fetal spine (Lawrence et al. 2024), kidney (Benjamin et al. 2024) and lung epithelium (Quach et al. 2024), while integration of scRNA-seq with CODEX mapping reveals cellular biogeography in human bone marrow niches (Bandyopadhyay et al. 2024). Simultaneously, Xenium has been utilized to characterize distinct regions of the mouse brain (Dennis et al. 2024; Kapustina et al. 2024; Marco Salas et al. 2025), enabling the identification of unique cell types and molecular interactions within tissues. This paves the way for investigating subcellular molecular mechanisms.

In conclusion, the integration of imaging and omics is evolving from a simple correlation of datasets into a deep, synergistic dialogue. We are advancing beyond a paradigm where omics provides a list of candidates that are then separately investigated by imaging. Instead, we are entering an era of continuous iterative cycle: imaging guides omics to meaningful targets, and omics reciprocally provides a comprehensive molecular dictionary to decipher the images. This iterative process of mutual guidance and validation is fundamentally reshaping our understanding of development and disease.

## Conclusions and perspectives

The integration of advanced imaging and single-cell omics has fundamentally reshaped developmental biology, transitioning the field from static observations to dynamic, spatially resolved systems biology. As we have highlighted, SRM and LSFM provide unprecedented views of subcellular and organism-scale dynamics, while single-cell and spatial omics reveal the molecular heterogeneity and geographic context underlying development. Critically, their synergistic combination—exemplified by resources such as Zebrahub (Kim et al. 2025), endoderm lineage tracing, and human fetal brain atlases—enables a holistic understanding of cell fate decisions, tissue patterning, and disease mechanisms (Table 3).

**Table 3:** Imaging and Single-cell Omics Integration in Developmental Biology

Technology	Strengths	Limitations	Representative Applications	Ref
SRM	Breaks diffraction limit; nanoscale visualization;	Limited molecular profiling; requires fluorescent labeling	Cytoskeletal remodeling; chromatin architecture; mitochondrial dynamics	Hübner et al. 2015; Qiao et al. 2023; Tsui et al. 2018
LSFM	Fast imaging; low phototoxicity; long-term 3D imaging; multi-scale capabilities	Lower resolution than SRM; limited deep-tissue penetration	Whole-embryo lineage tracing; organ morphogenesis; embryonic development	Agarwala et al. 2022; Belle et al. 2017; Daetwyler et al. 2025; Zhang et al. 2024
Single-cell Omics	Comprehensive molecular profiling; resolves cellular heterogeneity	Loss of spatial context; dissociation artifacts	Cell type discovery; lineage mapping; disease heterogeneity	Cao et al. 2019; Lang et al. 2023; Liang et al. 2021; Qiu et al. 2022; Sirkis et al. 2023; Wen et al. 2024
Spatial Transcriptomics	Preserves tissue architecture; maps gene expression in situ	Resolution platform-dependent; limited protein detection	Embryonic organogenesis mapping; cell–cell interaction studies	Abedini et al. 2024; Li et al. 2025b; Liu et al. 2022; Sampath Kumar et al. 2023; Wang et al. 2023; Wu et al. 2024; Xiao et al. 2024; Xie et al. 2025
Integrated Imaging with Omics	Links spatial dynamics with molecular states; enables multi-modal validation	Complex data integration; requires advanced computational analysis	Zebrafish embryonic development; HEC heterogeneity; brain development atlases	Kim et al. 2025; Nardone et al. 2024; Xia et al. 2023

Critically, integrating imaging with omics bridges the gap between cellular behavior and molecular mechanisms. Pioneering studies—such as Zebrahub (zebrafish), endoderm lineage tracing (mouse) (Li et al. 2025a), and human fetal brain atlases (Qian et al. 2025), demonstrate how combined approaches elucidate spatiotemporal dynamics of cell fate decisions, tissue patterning, and regenerative processes. This synergy not only resolves longstanding questions in development but also unveils disease-related spatial alterations (e.g., fibrosis and colitis).

The continuous optimization and synergistic integration of imaging and omics technologies are establishing an unprecedented perspective for resolving fundamental questions in developmental biology, solidifying this approach as the new research paradigm. This evolution transcends mere morphological description or molecular feature mapping, aiming for multi-faceted validation through the mutual integration and cross-verification of imaging and omics data. Looking ahead, the integration of real-time multi-omics imaging with predictive artificial intelligence (AI) models will be pivotal. This powerful combination will not only describe developmental events but will also enable the formulation of testable, computational models that can predict system-level responses to genetic or environmental perturbations. As these technologies advance, they will significantly deepen our understanding of life's most intricate processes and accelerate translational applications in regenerative medicine as well as in disease modeling and intervention.

The synergistic convergence of imaging and omics technologies—through deep integration and mutual validation—has profoundly transformed our understanding of developmental biology. Notably, recent breakthroughs in live-cell multi-omics imaging have achieved preliminary success in simultaneous profiling at single-cell resolution. Chen et al. demonstrated this through Live-seq—a method combining fluidic force microscopy (FluidFM) with enhanced low-input RNA-seq—which enables temporal transcriptomic recording while preserving cell viability, thereby directly linking a cell's ground-state transcriptome to its downstream phenotypic responses (Chen et al. 2022b). Nevertheless, substantial challenges persist. The path forward will be defined by synergistic advancements across three interconnected frontiers:

First, enhanced multi-omics methodologies must achieve truly simultaneous in situ profiling of multiple molecular layers within intact biological systems. A key foreseeable direction is the technical integration of live-cell transcriptomics (e.g., Live-seq) with LSFM. This would enable continuous recording of a cell's transcriptomic history while simultaneously visualizing its spatial behavior and division dynamics, directly coupling molecular causality with phenotypic outcome.

Second, sophisticated AI-driven analytical frameworks are imperative to manage the complexity of multidimensional datasets. We specifically foresee the application of deep learning architectures, such as graph neural networks and multimodal variational autoencoders, to perform seamless data fusion. These models can integrate spatial coordinates, temporal gene expression, and protein networks to build predictive models of cell fate decisions, forecasting how perturbations alter developmental trajectories.

Third, a paradigm shift toward live-cell multi-omics imaging is essential to transcend limitations of fixed-sample analysis. The power of multi-omics integration depends on AI to interpret its complexity, while live-cell imaging provides the temporal dimension that validates mechanistic insights. Moving beyond dissociated or preserved samples represents not merely a technical improvement, but a transformation in our capacity to observe life processes as integrated, dynamic systems in real time.

## Data Availability

Not applicable.

## Abbreviations

AI: Artificial Intelligence
CHD: Congenital Heart Disease
CLEM-SBFSEM: Correlative Light and Electron Microscopy with Serial Block-Face Scanning Electron Microscopy
CODEX: Co-Detection by Indexing
FluidFM: Fluidic Force Microscopy
HEC: Hematopoietic Endothelial Cell
HSPC: Hematopoietic Stem/Progenitor Cell
IMC: Imaging Mass Cytometry
LSFM: Light-Sheet Fluorescence Microscopy
MERFISH: Multiplexed Error-robust Fluorescence In Situ Hybridization
NGS: Next-Generation Sequencing
PALM: Photoactivated Localization Microscopy
PSF: Point Spread Function
SCoPE-MS: Single-Cell ProtEomics by Mass Spectrometry
scATAC-seq: Single-Cell Assay for Transposase-Accessible Chromatin with sequencing
scDNA-seq: Single-Cell DNA Sequencing
scRNA-seq: Single-Cell RNA Sequencing
SIM: Structured Illumination Microscopy
SRM: Super-Resolution Microscopy
Stereo-seq: Spatial Enhanced Resolution Omics-sequencing
STED: Stimulated Emission Depletion
STORM: Stochastic Optical Reconstruction Microscopy
TACIT: Target Chromatin Indexing and Tagmentation
UMAP: Uniform Manifold Approximation and Projection
